# Nrf2: A unifying transcription factor in the pathogenesis of Fuchs’ endothelial corneal dystrophy

**DOI:** 10.1016/j.redox.2020.101763

**Published:** 2020-10-16

**Authors:** Matthew Lovatt, Viridiana Kocaba, Dawn Jing Hui Neo, Yu Qiang Soh, Jodhbir S. Mehta

**Affiliations:** a Tissue Engineering and Cell Therapy Group, Singapore Eye Research Institute, Singapore; b Department of Cornea and External Eye Disease, Singapore National Eye Centre, Singapore; c Ophthalmology and Visual Sciences Academic Clinical Program, Duke-NUS Medical School, Singapore; d Netherlands Institute for Innovative Ocular Surgery (NIIOS), Rotterdam, the Netherlands

**Keywords:** Cornea, Fuchs' endothelial corneal dystrophy, Oxidative stress, Nrf2, ARE, antioxidant response element, CE/CECs, corneal endothelium/corneal endothelial cells, CHED, congenital hereditary endothelial dystrophy, DM, Descemet's membrane, DM1, myotonic dystrophy type 1, EK, endothelial keratoplasty, FECD, Fuchs' endothelial corneal dystrophy, FRDA, Friedreich's ataxia, HD, Huntingdon's disease, Keap1, Kelch-like ECH-associated protein 1, MEFs, mouse embryonic fibroblasts, Nrf2, Nuclear factor,erythroid 2 like 2, TNR, trinucleotide repeat

## Abstract

Nuclear factor, erythroid 2 like 2 (Nrf2), is an oxidative stress induced transcription factor that regulates cytoprotective gene expression. Thus, Nrf2 is essential for cellular redox homeostasis. Loss or dysregulation of Nrf2 expression has been implicated in the pathogenesis of degenerative diseases, including diseases of the cornea. One of the most common diseases of the cornea in which Nrf2 is implicated is Fuchs’ endothelial cornea dystrophy (FECD). FECD is the leading indication for corneal transplantation; and is associated with a loss of corneal endothelial cell (CEC) function. In this review, we propose that Nrf2 is an essential regulator of CEC function. Furthermore, we demonstrate that deficiency of Nrf2 function is a hallmark of FECD. In addition, we advocate that pharmacological targeting of Nrf2 as a possible therapy for FECD.

## 1 Introduction

Nuclear factor, erythroid 2 like 2, or Nrf2 is a transcription factor that regulates the expression of many genes encoding antioxidants. Nrf2 is highly conserved among aerobic species. Indeed, it has been hypothesized that Nrf2 may have evolved some 2 billion years ago, following a significant increase in levels of free oxygen in the earth's atmosphere, in what is called the ‘Great Oxidation Event’ [1]. However, whilst oxygen is utilized by most tissues in the body for cellular metabolism, a natural by product is the generation of reactive oxygen species (ROS) and free radicals, which are damaging to cell membranes and can induce DNA damage. Therefore, cells have adapted antioxidant defense systems which enzymatically reduce free radicals, for example, catalase, thioredoxins, peroxiredoxins and glutathione peroxidase [2].

The gene encoding Nrf2, *NFE2L2* was first isolated from erythroid cells in 1994 and, at that time, was thought to encode a basic leucine zipper transcription factor that regulates beta-globin expression [3]. However, Nrf2 deficient mice develop normally, demonstrating that Nrf2 is not essential for erythropoiesis [4]. Subsequently, it was discovered that Nrf2 binds to regulatory elements, termed antioxidant response elements (ARE) in DNA. The ability of Nrf2 to bind ARE provided the first suggestion that Nrf2 might control expression of genes encoding antioxidants, and protect against oxidative stress [5,6]. This was later correlated by the discoveries that Nrf2 deficient mice are highly susceptible to oxidative stress [7,8] and that Nrf2 regulates antioxidants vital for cytoprotection [9]. In addition, loss of Nrf2 function has been attributed to several neurodegenerative diseases in humans [10].

The cornea is metabolically active and exposed to ultraviolet (UV) radiation as well as pollutants and it is known to be under oxidative stress [11]. Therefore, oxidative stress has been recognised to be associated with certain corneal diseases.

Herein, we focus on the role Nrf2 plays in regulating homeostasis in the cornea with an emphasis on a late onset, blinding corneal disease known as, Fuchs endothelial corneal dystrophy (FECD). We propose that Nrf2 is central to the phenotypic and morphological changes apparent in FECD and that the pharmacological targeting of Nrf2 would be a valuable avenue for treatment of FECD.

## 2 Layers of the cornea

The cornea is the external tissue at the front of the eye which is crucial for visual clarity. It is an avascular, transparent structure, which provides the majority of the refractive power of the eye [12]. Cornea degeneration, dysfunction, damage or infection are the leading cause for corneal transplantation. Indeed, the cornea is one of the most transplanted tissues in the body [13]. However, globally the demand for transplant grade corneas vastly surpass the number of available donor corneas [13]. As an external organ, the eye is exposed to DNA damaging wavelengths of ultraviolet (UV) radiation. A function of the cornea is to absorb and protect the internal structures of the eye from harmful UV radiation [14].

**Fig. 1:**
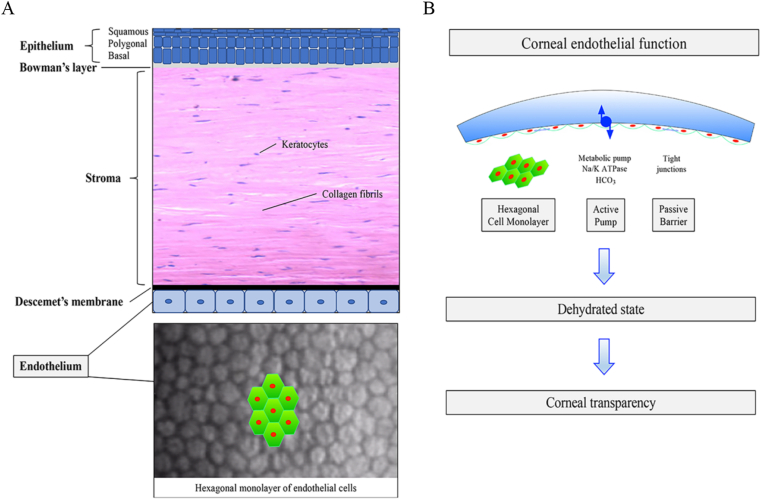
(A) Schematic of the cornea depicting the five main layers. The outer epithelial cell layer is separated from the stroma layer by a thin membrane termed Bowman's membrane. Embedded in the corneal stroma layer are keratocytes. The Descemet's membrane separates the stroma for a monolayer of hexagonal corneal endothelial cells. (B). Corneal endothelial cells (CECs) regulate corneal transparency by maintaining the stroma in a relative dehydrated state (deturgescence). Excess fluid is actively pumped out of the stroma by metabolic pumps such as Na^+^K^+^ATPase. However, the corneal endothelium is not impermeable and permits the passive diffusion of nutrients into the stroma thus maintaining proper corneal function.

A stratified, non-keratinizing epithelium forms the outermost layer of the cornea, followed by the Bowman's layer, which is an acellular condensation of collagen fibrils situated between the epithelial basement membrane and the underlying corneal stroma. The corneal stroma comprises a highly ordered array of collagen fibrils, extracellular matrix and water, embedded with stromal keratocytes, which are vital to maintain corneal transparency and wound healing (Fig. 1A). A thin basement membrane termed Descemet's membrane (DM) separates the stroma from the corneal endothelium (CE), which comprises a monolayer of corneal endothelial cells (CECs); (Fig. 1A). CECs are arranged in a hexagonal-like orientation to maximize surface density, (Fig. 1A). The CE is in direct contact with the aqueous humor and plays a vital role in regulating hydration and clarity of the cornea. CECs express proteins involved in active fluid transport, such as Na^+^K^+^-ATPase and SLC4A11 located in the basolateral cell membrane, which dehydrate the corneal stroma [15]. Glucose transporters located on both basolateral and apical aspects of CEC cell membranes ensure a constant glucose flux from the aqueous into the corneal stroma. While the presence of tight junctions between CECs prevents passive intercellular transit of fluids, they do not exist in continuous bands, which thus allows for the passive ingress of fluid from the aqueous into the corneal stroma.

A complex interplay and dynamic equilibrium between these active and passive mechanisms driving corneal stroma hydration status is essential to maintain corneal stroma deturgescence within a tight and specific range, critical for achievement of corneal transparency. (Fig. 1B). The human cornea lacks significant regenerative capacity, and CECs gradually decline with age, especially in the early post-natal period [16]. Modest CEC loss can be compensated for by the spreading and stretching of CEC borders. However, once CEC loss exceeds a critical threshold, corneal endothelial pump failure ensues, and cornea clarity is lost [12]. The resultant cornea clouding and blindness is irreversible without medical and/or surgical intervention (Fig. 1B). CEC loss may also occur secondary to trauma, viral infections, iatrogenic causes, for example, cataract surgery causing pseudophakic bullous keratopathy) and corneal dystrophies (for example, FECD and Congenital Hereditary Endothelial Dystrophy, CHED).

## 3 Fuchs endothelial corneal dystrophy

**Fig. 2:**
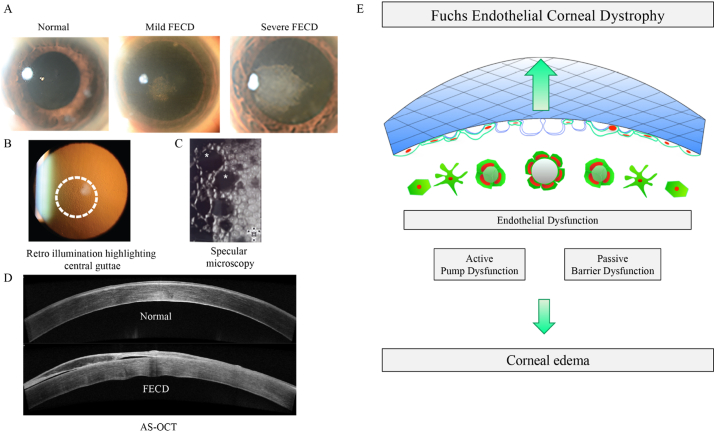
Clinical features of FECD include scar formation, the presence of guttae on DM, CEC loss and corneal edema. FECD is visualised and staged by (A) slit lamp microscopy, (B) retroillumination (C) specular microscopy & (D) anterior segment optical coherence tomography (AS-OCT). The presence of guttae causes the appearance of dark patches (* in panel C) which causes large gaps in the monolayer of CECs. (E) Schematic demonstrating how the presence of guttae disrupts the monolayer of corneal endothelial cells resulting in loss of CEC function. Corneal endothelial dysfunction causes painful corneal swelling and ultimately loss of vision.

Late onset FECD is the most common cause of CEC dysfunction. FECD was first described over 100 years ago [17]. Clinically, FECD is characterised by the progressive degeneration of CE with a visible decrease in CEC density and abnormal CEC morphology (Fig. 2). Gradual CE dysfunction leads to corneal edema and loss of corneal clarity. A hallmark of FECD pathogenesis, is the presence of excrescences of anomalous extracellular matrix (ECM), termed guttae, deposited on the DM. In early stages of FECD, guttae are localized to the central cornea, but they can evolve to involve a much larger area extending to beyond the central 4–6 mm of the cornea. The most commonly employed clinical criteria for grading FECD, initially proposed by Krachmer [18] in 1978, was an anatomical grading system which placed great emphasis on the distribution and density of guttae, with the presumption that corneal edema only occurs in the most advanced stage of the disease. Clinically, FECD is assessed through image analysis (Fig. 2A). This includes slit lamp microscopy with retroillumination [19], which assesses the stage of FECD as well as the number and distribution of guttae. In addition, the corneal endothelium and guttae can be directly imaged through non-invasive specular microscopy. Anterior segment optical coherence tomography (AS-OCT) and scheimpflug imaging [20]that allows analysis of secondary corneal changes i.e. edema that is known to occur in FECD. Together these imaging tools allow accurate FECD diagnosis and clinical prognosis.

The 3-dimensional structure of the guttae in part contributes to the disease, as large guttae physically disrupt the CE monolayer (Fig. 2B) [21]. Hence, it has been demonstrated that when normal CECs are seeded onto FECD-DM, the largest guttae induce cellular changes and result in apoptosis of the CEC [22]. However, it is not known if guttae directly trigger CEC stress and cell death or whether CEC stress stimulates guttae formation [17]. Ultimately, late stage FECD results in loss of CE function which leads to corneal edema, resulting in painful swelling of the cornea, loss of visual acuity and if left untreated blindness.

Despite clinical and scientific advances in understanding the pathogenesis of FECD, treatments of FECD are limited to direct cellular replacement. Currently, surgical intervention in the form a corneal transplantation is the only suitable therapy for advanced stage FECD.

Globally the incidence and prevalence of FECD differs greatly. However, in the U.S approximately 4% of the population over the age of 40 years are diagnosed with FECD. Interestingly, females are more affected than males [23]. Typically, Asian populations show fewer cases of FECD [24,25]. This may well be due to certain genetic loci associated with pathogenesis of FECD (see below).

The inheritance and genetics of FECD are complicated. Several different loci have been implicated in FECD. This includes: TCF8 [26], SLC4A11 [27], LOXHD1 [28], KANK4, LAMC1 and ATP1B1 [29]. Additionally, an expanded CTG trinucleotide repeat (TNR) within an intron of the TCF4 gene has been demonstrated to be prominently associated with FECD [[30], [31], [32], [33]]. However, globally amongst different ethnic groups the frequency of FECD patients harboring the repeat sequence vastly differs. For example, it is estimated that amongst the Caucasian population with FECD the prevalence of repeat sequence is around 70%, compared to both Asian and the African American population where the prevalence is much lower (~25–50%) [34]. Therefore, not all FECD can be attributed to CTG repeat expansion., FECD is more prevalent in females than in males with LAMC1 thought to confer greater risk in females; whilst TCF4 increased risk in males [29].

Surgical intervention, in the form of endothelial keratoplasty (EK), is currently the best option for restoring vision in advanced FECD. In a standard EK surgery, such as Descemet Membrane Endothelial Keratoplasty (DMEK), diseased host DM and CE is first stripped, following by allogenic transplantation of a cadaveric donor DM and CE complex, in order to restore the host corneal endothelial pump [35,36]. Stripped DM and CE tissue from FECD patients has been an excellent source of material for laboratory analysis, particularly as FECD-CECs can be isolated attached to their natural DM substrate. In addition, protocols for isolating, expanding and immortalizing normal as well FECD-CECs has allowed long term cultures of CECs to be established to further probe the pathogenesis of FECD [[37], [38], [39], [40], [41]]. For example, CE attached to DM from FECD patients undergoing surgery have been compared to normal CE/DM by PCR based array analysis [42,43]. This data has demonstrated global mRNA changes in FECD including, an imbalance in genes known to regulate oxidative stress that are also targets of Nrf2 [42,43]. Importantly, Nrf2 has also been reported to be downregulate in FECD [44]. Therefore, above and beyond any hereditary genetic factors, oxidative stress and an imbalance in antioxidants play a significant role in the pathogenesis of FECD. As Nrf2 is central to the regulation of oxidant-antioxidant poise together, and in addition to genetic susceptibility loci, it undoubtedly implicates Nrf2 in the pathogenesis of FECD.

We will discuss in greater detail particular aspects of Nrf2 expression, regulation and interacting partners, highlighting possible functions in CE and FECD.

## 4 Regulation of Nrf2

**Fig. 3:**
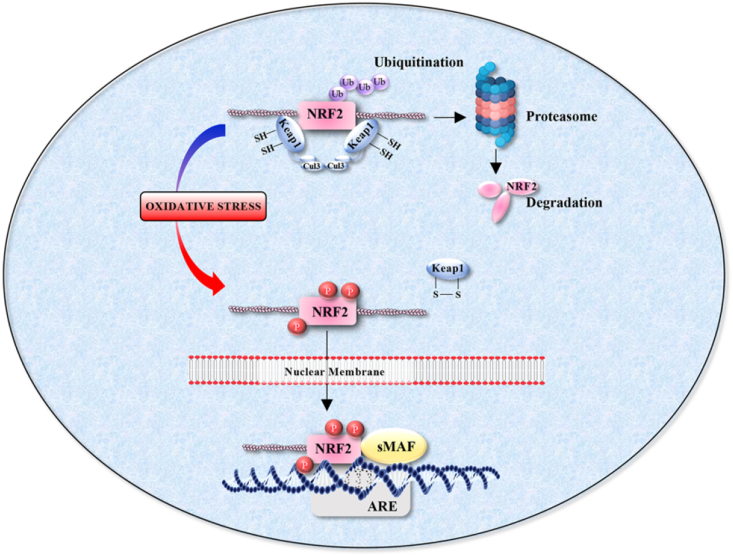
The Nrf2 pathway. In the absence of oxidative stress the basal levels of Nrf2 are very low. Nrf2 is sequestered in the cytoplasm through its association with the Kelch-like ECH-associated protein 1 (Keap1) which constitutively targets Nrf2 for ubiquitin dependent protein degradation. Oxidative stress modifies cysteine residues in Keap1 resulting in the release of Nrf2 from ubiquitination. Subsequently, Nrf2 is able to translocate to the nucleus and induce transcription of genes which contain the binding site, antioxidant response element (ARE) in their promoter.

Under basal conditions Nrf2 levels are low as Nrf2 is constitutively ubiquitinated by the cysteine rich Kelch-like ECH-associated protein 1 (Keap1) [9]. As a scaffold component of the Cullin 3-based ubiquitin E3 ligase, Keap1 targets Nrf2 for proteasomal degradation (Fig. 3). Consequently, basal Nrf2 levels are kept low. Keap1 acts as an oxidative stress sensor. Reaction of critical cysteine residues in Keap1 with H_2_O_2_ results in a conformational change in its structure. Subsequently, Keap1 is rendered inactive thus allowing stabilisation of Nrf2 [45]. Activated Nrf2 translocates to the nucleus and heterodimerizes with small maf (sMaf) protein family. Nrf2-sMaf heterodimers bind to regulatory elements in DNA and facilitate the transcription of a myriad of genes associated with de-toxification and cytoprotection. In addition, putative sites of phosphorylation in Nrf2 suggest additional regulation of Nrf2 function by certain kinases. However, the exact role of phosphorylation in Nrf2 function has not been fully elucidated [46]. The *cis*-regulatory elements for Nrf2 binding are comprised of a 41 base pair recognition sequence termed antioxidant response elements (ARE). Interestingly, the levels of Keap1 have been demonstrated to be elevated in FECD [44]. In addition, the Parkinson associated disease protein PARK7 (also known as DJ-1) assists in the stabilisation of Nrf2 and has been implicated in the pathogenesis of FECD as DJ-1 levels are severely reduced in FECD tissue [44]. Moreover, siRNA targeting of DJ-1 in transformed CE cell lines impairs Nrf2 translocation to the nucleus and the defective upregulation of the Nrf2 target gene NQO1 [47]. Loss of DJ-1 sensitised CE cell lines to UV induced apoptosis, consistent with a role for Nrf2/DJ-1 as an antioxidant [47].

In addition to regulating Nrf2 levels, Keap1 has been demonstrated to interact with a number of other proteins and therefore play roles in addition to a repressor of Nrf2 [48]. Age dependent decrease in Nrf2 with concomitant increase in Keap1 expression has been reported to occur in epithelial cells of the lens [49]. This was particularly evident from lenses isolated from donors above the age of 65 years and occurred at both mRNA and protein level and correlated with a demethylation in the keap1 promoter [49]. FECD is predominantly a disease of late onset and advanced age is a known risk factor for developing FECD [17]. However, it has not been determined if Keap1 levels in CEC increase with age and whether or not this proceeds loss of Nrf2 and phenotypic changes associated with FECD.

### 4.1 Nrf2 target genes

To identify genes regulated by Nrf2, mouse embryonic fibroblasts (MEFs) derived from either Keap1 or Nrf2 knockout mice have been studied. Loss of Keap1 resulted in constitutive Nrf2 activation. Surprisingly, whilst homozygous Keap1 null mice survive only until weaning age [50], Nrf2 deficient mice are viable [4]. Chromatin-immunoprecipitation (ChIP-Seq), DNA sequencing and microarray have been utilized to identify >600 putative genes containing ARE that are upregulated in the absence of Keap1 and downregulated in the absence of Nrf2 [51]. Employing human lymphoblastoid cells Chorley et al. performed ChIP-seq analysis on cells treated with the Nrf2 activator sulforaphane [52]. Similar to MEFs over 849 genes were identified to contain at least one ARE, upstream of the transcription start sites of the genes. A comparison between this and the MEF study revealed an overlap of 110 genes [52]. The large overlap between the two studies suggest that Nrf2 target genes are conserved in both mouse and human cells. The identification of genes with ARE explains the multifaceted role for Nrf2 in the regulation of oxidative stress and, it is known that Nrf2 also regulates expression of genes not directly linked to oxidative stress. For example, mitochondria bioenergetics, the unfolded protein response (UPR), iron metabolism, proteasome activity and focal adhesion. ChIP-seq analysis of the lung carcinoma cell line, A549 has revealed that a number of genes associated with focal adhesion are regulated by Nrf2, including LAMC1, which has previously been implicated in FECD [29,53].

Nrf2 controls the expression of many cellular components including, glutathione (GSH) synthesis, enzymes involved in detoxification and NADPH regeneration as well as heme and iron catabolism. We have summarised relevant Nrf2 targets based on their association to CEC and/or FECD in Table 1.

**Table 1:** Summary of genes regulated by Nrf2 relevant to CEC and FECD.

Gene	Protein name	Functional significance to CEC	Identified as Nrf2 target from: (Cell/tissue)	Confirmed ARE Reference(s)
NQO1	NAD(P)H quinone oxidoreductase 1	Targeted reduction in CEC sensitises CEC to oxidative stress [54] and expression downregulated in FECD [55]	Multiple cell type	[51,52,[56], [57], [58]]
Prdx1/6	Peroxiredoxin 1 and 6	Expression down regulated in FECD [59,60]. Prdx1 regulates lipid peroxidation in CEC [60]	MEFs	[51]
SOD	Superoxide dismutase	SOD mRNA levels reduced in FECD [42,43]	Lymphoblastoid cell lines	SOD1 [52]
ABCB6	ATP binding cassette subfamily B member 6	Linked to generation of and catabolism of heme Indirectly via ferroptosis [61]	Multiple cell types	[51,52,57,62]
FTH1 TL	Ferritin (heavy & light chain)			
FECH	Ferrochelatase			
HO1	Heme Oxygenase			
SLC48A1	Heme transport			
BLVRA/B	Bilverdin reductase A&B			
SLC7A11	Cystine/glutamate antiporter xCT	mRNA level regulated by Nrf2 in human CEC line B4G12 [60]	Lymphoblastoid cell lines, MEFs	[52,63]
GPx4	Glutathione peroxidase 4	GPx4 regulates lipid peroxidation in B4G12-CEC [64]	MEFs	[63]

## 5 The role of oxidative stress in the pathogenesis of FECD

A common etiological factor in the pathogenesis of FECD is the involvement of oxidative stress. A number of genes involved in regulating oxidative stress have been demonstrated to be down regulated compared to normal CECs [42]. This includes the transcriptional down regulation of the family of redox sensors peroxiredoxins, a finding subsequently verified by analysis of protein extracted from FECD patient samples [59,60]. As a large proportion of dysregulated genes contain an ARE, hence it suggests Nrf2 is central to regulating oxidative stress in CECs. Consistent with this is the evidence that Nrf2 protein levels are significantly reduced in FECD-CECs compared to normal controls [42]. Loss of Nrf2 and thus the Nrf2 regulated oxidative stress response in FECD suggests that over an individual's lifetime the constant UV exposure accentuates CEC death to a pathological level. Evidence for which has been obtained from both *in vitro* studies [65], as well as an *in vivo* animal model of UV induced cornea damage [23]. Cultures of human CECs exposed to UVA have been demonstrated to upregulate both Nrf2 mRNA and result in the translocation of Nrf2 to the nucleus resulting in induction of the Nrf2 regulated genes NQO1 and HO-1 [65]. However, prolonged UVA exposure also triggered caspase dependent apoptosis [65]. Importantly, UVA exposure on the mouse cornea induces guttae-like deposits within the CE, together with morphological changes similar to FECD. Notably, the corneas from female mice are more susceptible to UVA, compared to male mice, thus recapitulating the sex differences apparent in FECD [23]. Interestingly, the estrogen metabolizing enzyme CYP1B1 is upregulated by UVA more prominently in CE derived from female mice compared to CE derived from male mice. Furthermore, CYP1B1 has demonstrated to be upregulated in human FECD tissue samples [23]. However, CYP1B1 can be regulated by NRF2 [51]. Therefore, exactly how loss of Nrf2 in FECD results in CYP1B1 upregulation is unclear. Perhaps loss of Nrf2 in FECD causes aberrant activation of another CYP1B1 regulator, aryl hydrocarbon receptor (AHR) which binds to a xenobiotic response element in CYP1B1 [66].

Increased apoptosis has been reported in CE isolated from FECD patients [42,67,68]. Moreover, CECs isolated from FECD patients have been demonstrated to be more susceptible to apoptosis induced by oxidative stress inducing agents such as *tert*-butyl hydroperoxide [37]. Apoptosis can be triggered through cytokine mediated signal transduction such as FAS and TNF triggered cell or through cells sensing stress through mitochondrial dysfunction or ER stress and the unfolded protein response.

### 5.1 Nrf2 and cytoprotection

The cell contains many enzymes dedicated to redox homeostasis. Many of which are regulated by Nrf2 (Table 1). The prototypical Nrf2 target, NQO1 is critical for cytoprotection and importantly has been demonstrated to be involved in the pathogenesis of FECD. Treatment of CEC with the quinone menadione induces cell damage which mimics the cellular changes seen in FECD. Menadione is reduced by NQO1. Utilizing a CEC line and targeting expression of NQO1 it has been demonstrated that NQO1 deficient cells are hypersensitive to menadione, suggesting Nrf2 mediated NQO1 expression is required to regulate the response to menadione [54,69]. Moreover, loss of NQO1 expression has been reported in CECs from FECD patients [55]. As noted above, FECD is more prevalent in females than males. Moreover, NQO1 is linked to estrogen metabolism and is known to remove genotoxic metabolites of estrodiol. Furthermore, loss of NQO1 in FECD exacerbates the response to estrogen genotoxicity [55].

### 5.2 Nrf2 and peroxiredoxins

The highly conserved and ubiquitous family of redox sensors peroxiredoxins (Prdx) are a family of antioxidants capable of reducing peroxides such as H_2_O_2_ and lipid peroxides [70]. Mammalians express six Prdx isoforms which differ in subcellular localisation [70]. As Prdx are constitutively expressed at high concentrations knowledge regarding their function has focused on their enzymatic activities rather than regulation of their expression. However, Nrf2, ChIP-seq data sets have revealed that both Prdx1 and Prdx6 are regulated by Nrf2 binding AREs in their promoter regions [51,52]. Transcriptional down regulation of Prdx 1, Prdx2, Prdx 5 and Prdx 6 has been reported in CE from FECD tissue [42]. In addition, proteomic analysis has revealed that Prdx2, Prdx3 and Prdx5 are downregulated in FECD [59]. Furthermore, evidence that Prdx1 expression is lost in CE from FECD patient derived tissue has also been demonstrated [60]. Targeting Nrf2 with siRNA in the CEC line B4G12 reduces Prdx1 mRNA levels and loss of both Prdx1 and Nrf2 was demonstrated to affect cumene hydroperoxide induced lipid peroxidation and cell viability [60]. In addition, Prdx6 has been implicated in regulation of mitochondrial membrane potential function, and loss of Prdx6 in B4G12 rendered B4G12-CEC more susceptible to cell death [71]. These data suggest that loss of Nrf2-Prdx axis may further contribute to the overall imbalance in oxidative stress apparent in FECD.

### 5.3 Nrf2 and mitochondria

Mitochondrial dysfunction is thought to play a prominent role in the pathogenesis of FECD [72]. Mitochondria are rich in CEC and are thought to be essential for providing the cellular energy required to maintain the pump-barrier function of CE. Loss of mitochondrial, superoxide dismutase 2 (SOD2) and an increase in mitochondrial DNA damage in FECD tissue was the first evidence that mitochondria in FECD may be affected [42]. The synthetic quinone, menadione (MN) generates intracellular mitochondrial superoxide and elevates intracellular ROS. The Nrf2 target gene, NQO1 metabolizes MN. Therefore, as noted above loss of Nrf2/NQO1 renders CE more sensitive to MN [69]. Furthermore and as discussed above, menadione induced mitochondrial depolarization has been demonstrated to affected by loss of another Nrf2 target, Prdx6, resulting in hypersensitive to menadione in the human CEC line B4G12 [71]. Defects in mitochondria in FECD include morphological change, loss of mitochondrial mass and absolute mitochondrial numbers, as well as loss of mitochondrial membrane potential [73]. Selective degradation and removal of damaged mitochondria, or mitophagy is controlled by the PINK1/Parkin pathway which has been suggested to be elevated in FECD along with an increase in mitophagy [74]. The PINK1 (PTEN-induced putative kinase 1) gene contains putative ARE regulatory elements in its promoter region and Nrf2 has been demonstrated to active PINK1 transcription in human neuroblastoma cell lines [75]. Moreover, loss of Nrf2 contributes to mitochondrial dysfunction as several features of mitochondrial function are disrupted in Nrf2 knockout mice [76,77]. For example, mouse embryonic fibroblasts isolated from Nrf2 deficient mice have reduced mitochondrial membrane potential and decrease mitochondrial respiration as judged by a decrease in NADH leading to reduced ATP synthesis [76].

### 5.4 SLC4A11 and Nrf2

SLC4A11 is a membrane transporter protein expressed on the basolateral surface of CEC, that contributes to corneal hydration as well as functioning as an adhesion molecule anchoring CECs to DM [78]. Several mutations in SLC4A11 have been described in FECD [27]. A large majority of mutations cause dysfunctional trafficking of SLC4A11 to the cell surface [79], resulting in cell stress reminiscent of UPR. Furthermore, ER retaining mutants of SLC4A11 have conferred an increased sensitivity to *tert*-butyl hydroperoxide (tBHP) when overexpressed in cell line [80]. In addition, siRNA mediated depletion of SLC4A11 also de-sensitised cells to oxidative stress inducing agents such as *tert*-butyl hydroperoxide (tBHP) [81]. Surprisingly, loss of SLC4A11 reduced Nrf2 mRNA levels and affected induction of NQO1 following tBHP treatment [81]. However, exactly how loss of SLC4A11 impacts Nrf2 is not known.

### 5.5 Unfolded protein response and Nrf2

The presence of misfolded proteins in the lumen of the endoplasmic reticulum (ER) is toxic to cells unless they are removed. The unfolded protein response (UPR) mechanism ensures removal of misfolded proteins. The UPR pathway is highly conserved and involves ER resident signalling components such as, activating transcription factor 6 (ATF6), inositol requiring enzyme 1 (IRE1) and double-stranded RNA-activated protein kinase (PKR)-like ER kinase, (PERK). The ER chaperone protein GRP78 binds misfolded proteins for further processing as well as activating IRE1, ATF6 and PERK. Activated ATF6 translocates to the Golgi and is proteolytically cleaved allowing a domain of ATF6 to enter the nucleus to serve as a transcription factor for the induction of UPR specific genes [82]. IRE1 possess kinase and endonuclease activity. Activated IRE1 removes an intron for the X-box binding protein (Xbp1) resulting in the unconventional splicing of Xbp1. Spliced Xbp1 acts as a transcription factor to upregulate genes involved in UPR [82]. PERK functions to attenuate any further translation of proteins via phosphorylation of the translation initiation factor eukaryotic initiation factor 2 (elF 2) [83].

Electron microscopy and immunofluorescence assessment of FECD corneas revealed abnormal ER morphology and upregulation of markers associated with the UPR response [84,85]. This includes the presence of GRP78 positive aggresomes in FECD CE [85]. Treatment of immortalized FECD as well as control CEC lines with the ER stress inducing compound, thapsigargin, results in the elevated activation of PERK and IRE1 in FECD cells compared to control [85]. This data suggest that increased ER stress in FECD may contribute to increased apoptosis and pathogenesis of FECD.

Interestingly, PERK has been demonstrated to stimulate expression of Nrf2 [86]. For example, in the mouse embryonic fibroblast cell line, NIH-3T3, ER stress induced Nrf2 translocation and expression of luciferase reporter constructs containing a functional ARE in a PERK dependent fashion [86]. Moreover, in the absence of Nrf2 MEFs fail to induce expression of the Nrf2 target gene the catalytic subunit, glutamate-cysteine ligase, (GCLC), following thapsigargin treatment [86]. In turn, Nrf2 upregulates genes involved with reducing the oxidative burden specifically in the ER [87]. This includes several gene products related to enzymes regulating glutathione (GSH) metabolism which is essential for maintaining physiology of the ER [87,88]. Taken together it is suggested there is significant crosstalk between ER stress, UPR and oxidative stress signalling pathways [89].

### 5.6 Nrf2, iron, lipid peroxidation and ferroptosis

Oxidative stress can be detrimental to all cellular organelles and membranes. Lipids, typically polyunsaturated fatty acids (PUFA) within cellular membranes can be damaged by oxidative stress resulting in lipid peroxidation. Extensive lipid peroxidation will result in loss of membrane integrity. Furthermore, lipid peroxidation generates aldehydes such as 4-hydroxynonenal (4-HNE) and malondialdehyde (MDA) which are toxic to cells and can lead to DNA damage [90]. Lipid peroxidation appears to trigger cell death via pathways distinct from the classical caspase dependent apoptotic pathways. This novel pathway was coined ferroptosis [91]. Ferroptosis was defined as an iron dependent, non-apoptotic, lipid peroxide driven cell death. Notably, Nrf2 is required at several stages of ferroptosis as several proteins involved in ferroptosis are known Nrf2 targets (Table 1) [61]. Moreover, ferroptosis is related to ER stress and UPR as both are regulated by cysteine/gluthamate transport and production of GSH and lipid peroxidation can occur in the ER [92].

Although iron is an essential element and cofactor required for many biological processes, under certain circumstances iron is capable of generating toxic hydroxl radicals (OH-) through the reaction with endogenously produced H_2_O_2_ (Fenton reaction). Therefore, the available pool of iron needs to be tightly regulated. A number of Nrf2 targets include genes that regulate either regulate iron metabolism or the synthesis, catabolism and degradation of heme (Table 1). The role of Nrf2 in regulating iron in biological processes has been expertly reviewed elsewhere [93]. Iron is required for ferroptosis: OH-radicals generated by the Fenton reaction can generate lipid peroxides. Moreover, ferroptosis can be effectively inhibited by iron chelators [91]. In addition, ferroptosis involves the Nrf2 transcriptional targets GPX4 and SLC7A11 [91,94] (Table 1). SLC7A11 encodes a subunit of the cystine/glutamate transporter vital for the generation of GSH. In turn, GPX4 utilises GSH to reduce lipid peroxides. Hence, Nrf2 regulates lipid peroxidation/ferroptosis via SLC7A11 and GPX4. Moreover, there is evidence that the level of Nrf2 can regulate ferroptosis in certain cell lines [95,96]. However, to date neither GPX4, SLC7A11 expression nor has ferroptosis been studied in relation to FECD. In the corneal endothelial cell line B4G12 (B4G12-CEC), depleting Nrf2 renders cells more susceptible to lipid peroxidation induced by cumene hydroperoxide (CH) with a concomitant decrease in SLC7A11 mRNA levels [60]. Interestingly, basal Prdx1 mRNA levels were reduced by loss of Nrf2 expression [60]. However, treatment of Nrf2 deficient B4G12 cells with CH largely restored Prdx1 expression. This data conflicts with previous publications describing Prdx1 regulation by Nrf2 in mouse macrophages [97], and cancer cell lines [52]. However, these studies did not look at the response to CH. Targeting Prdx1 by siRNA in B4G12-CEC similarly rendered cells more susceptible to lipid peroxidation resulting in a decrease in cell viability [60]. As previously discussed, both Prdx1 and Nrf2 are decreased in FECD. It is thus tempting to speculate the lipid peroxidation induced ferroptosis pathway might contribute to the pathogenesis of FECD.

The Nrf2 target GPX4 is an essential regulator of ferroptosis as it protects against damaging lipid peroxidation [94]. Utilizing B4G12-CECs two groups have independently targeted GPX4 with siRNA [60,64]. Both studies confirmed GPX4 expression in B4G12-CEC and demonstrated enhanced lipid peroxidation in response to oxidative stress [60,64]. In summary there is substantial evidence to suggest that in addition to apoptosis, Nrf2 may regulate non-apoptotic cell death such as ferroptosis. The role of ferroptosis in age dependent neurodegenerative diseases is an emerging field. Inhibitors of ferroptosis have demonstrated protection in certain models of degenerative disorders including Parkinson's and Alzheimer's [98]. However, the significance of ferroptosis in the pathology of FECD needs further exploration.

## 6 Nrf2 and trinucleotide repeat disorders

Trinucleotide repeat (TNR) disorders are genetic diseases caused by expansion of trinucleotides repeats. They include: myotonic dystrophy type 1 (DM1), caused by a CTG expansion in the DMPK1 gene, Huntingdon's disease (HD), caused by a CAG expansion in the huntingtin gene (HTT) and Friedreich's ataxia, caused by GAA expansion in frataxin (FXN). Generally, TNRs cause unstable, toxic RNA and protein thus disrupting normal cellular function. By far the most common mutation associated with FECD is an unstable CTG expansion in the intron of TCF4. Exactly how the TNR causes pathological damage to CE is not clear. Based on other TNR diseases such as myotonic dystrophy type 1 (DM1), the prevailing view is that the triplet expansion in TCF4 produces the expression of toxic RNA resulting in discrete nuclear RNA foci that sequester the mRNA splicing factor, MBN1 [99]. Consequently, defective splicing is toxic to the cell. Evidence for toxic RNA foci has been described in CE from FECD but not in non-FECD controls [100]. In addition, expression of repeat associated non-ATG (RAN) translation of TCF4, has been detected in CE from FECD patients [101]. RAN translation is bidirectional and can result in multiple reading frames producing toxic proteins which could further contribute to disease via increased ER and oxidative stress. TNR diseases have been associated with oxidative stress. Furthermore, neural stem cells from HD harbouring a CAG expansion negatively impact Nrf2 signalling [102]. The activation of Nrf2 with the Nrf2 activating compound MIND4-17 failed to induce NQO1 expression in neural stem cells with repeats; however, removing the repeat sequence, via homologous recombination, restored the ability of MIND4-17 to induce NQO1 [102]. This data suggests that the repeat sequence exacerbates pathology through the inhibition of Nrf2 [102]. Furthermore, oxidative stress has been demonstrated to increase CAG repeats in embryonic stem cells derived from a transgenic mouse model of HD [103]. The transgenic mouse model of HD demonstrated age-dependent somatic expansion of the TNR, a process which is accelerated because of oxidative DNA damage [104].

Friedreich's ataxia (FRDA), an autosomal recessive neurodegenerative condition is known to result from a GAA repeat in the mitochondrial protein frataxin. Predominantly the spinal cord, brain and heart are affected in FRDA. Dysfunctional mitochondria, oxidative stress, including increased ROS and lipid peroxidation are key factors in the pathogenesis of FRDA. One potential therapeutic target for FRDA is Nrf2. Under oxidative stress conditions Nrf2 fails to translocate to the nucleus in FRDA fibroblasts [105]. In addition the induction of Nrf2 target transcripts are severely affected by loss of Nrf2 [105]. The level of Keap1 is significantly higher in FRDA fibroblasts [106,107] suggesting that loss of Nrf2 activity may be due to elevated Keap1 expression. The synthetic compound omaveloxolone (RTA-408) is currently under clinical investigation for treatment of FRDA [108,109]. Omaveloxone is an Nrf2 activator demonstrated to rescue mitochondrial defects in animal models of FRDA as well as in FRDA patient fibroblasts [109] Interestingly, omaveloxone is also under trial to prevent corneal endothelial cell loss in patients undergoing cataract surgery (www.clinicaltrials.gov).

The penetrance of TNR in diseases such as HD and FRDA is unequivocal. FECD is atypical in that not all patients with FECD have the TCF4 CTG repeat. However, >40 CTG repeats in patients, has been shown to correlate with progression and severity of disease thus increasing the likelihood of transplantation [[110], [111], [112], [113], [114], [115]]. Similar to HD, FECD is late onset, typically affecting patients >40 years old. It is not known if the CTG repeat in TCF4 expands with age, furthermore, it has not been determined whether oxidative stress and principally the loss of Nrf2 might increase the expansion rate.

## 7 Therapeutic Nrf2 agonists

The pharmacological targeting of Nrf2 through small molecules is being explored for several neurodegenerative diseases. Generally, activators of Nrf2 target critical oxidative stress sensing cysteine residues in Keap1 [116]. As previously mentioned the Nrf2 activator Omalarone (RTA 408) is being used as a possible therapeutic for FRDA [108]. Small molecules like Omalarone target a critical cysteine residue (C151) in Keap1 [117]. Similarly, a C151 modifying compound for HD was identified through library screening which led to the identification of MIND4-17 [102]. MIND4-17 was demonstrated to be a highly potent Nrf2 activator. MIND4-17 has demonstrated promising results in restoring oxidative stress and neuroinflammatory induced defects in both mouse and human cell models of HD [102]. Sulforaphane is a natural compound found in cruciferous vegetables such as broccoli and cabbage has potential therapeutic application; as it possess cytoprotective effects [116]. Sulforaphane is another Cys-151 modifier that has been suggested as a therapy for FECD as it has been demonstrated to reduce apoptosis of FECD-CEC through the re-establishment of the Nrf2 pathway [118]. A number of molecular cellular defects have been described FECD, which we believe are showing the downstream effects of dysregulation in Nrf2. The use of Nrf2 activators in other diseases strongly suggests that pharmacological targeting of Nrf2 could be a worthwhile avenue of research for FECD. However, given the lethality of Keap1 knockout mice presumably due to hyper-Nrf2 activity. The timing of Nrf2 activators will need to be tightly controlled and ideally applied locally.

## 8 Conclusions and future directions

**Fig. 4:**
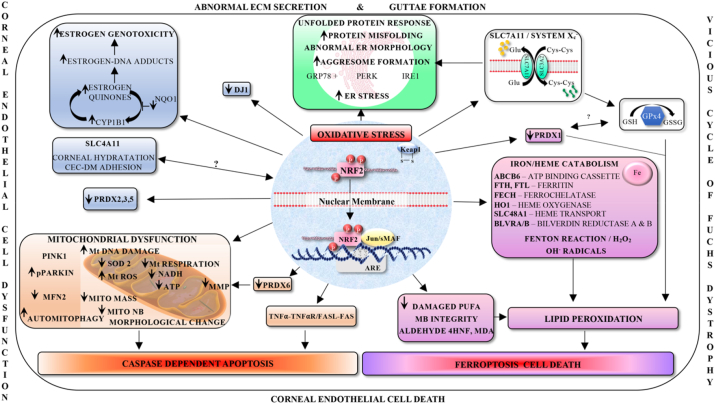
Schematic depicting roles for Nrf2 in regulating the functioning of corneal endothelial cells (CECs). We hypothesise that the phenotypic changes in FECD can be attributed to loss of Nrf2 expression in CECs. Subsequently, a multitude of defects including, loss of cytoprotection, mitochondrial dysfunction and increased lipid peroxidation culminates in irreversible cell death. Therefore, the use of pharmacological targeting to restore Nrf2 expression should be explored as a therapy for FECD.

The underlying phenotype in FECD is an imbalance in oxidative stress. Central to this is a significant decrease in the expression of Nrf2. Loss of Nrf2 activity triggers a multitude of responses culminating in mitochondrial dysfunction, DNA damage, excessive lipid peroxidation and ultimately cell death. Additionally, both endogenous (genetic factors) and exogenous factors (environmental factors such as UV light) perpetuates the vicious circle of FECD [17] (Fig. 4). Death of CE is permanent as CE lacks regenerative capacity. Patients harbouring expanded CTG repeat sequence in their TCF4 locus have an additional burden, which further predisposes the advancement of disease pathogenesis resulting in an increase need for transplantation. Given the global shortage of transplant grade cornea tissue there is a need for alternative therapies to slow progression of FECD.

Improvements in FECD grading and diagnosis will be essential for early detection and intervention. For the most part, FECD has been studied using late stage ex vivo tissue or transformed cell lines. Therefore, there are several important questions that need to be addressed to understand early detection of FECD. This includes whether loss of Nrf2 expression in FECD is a cause or consequence of increased sensitivity to oxidative stress. Furthermore, it is not known why some patients have expanded CTG repeats whilst others do not.

In summary, we propose that early diagnosis of FECD together with the pharmacological targeting of Nrf2 should be actively explored as a therapy for FECD.

## Declaration of competing interest

The authors declare no competing interests.

## References

[1] Gacesa R., Dunlap W.C., Barlow D.J., Laskowski R.A., Long P.F. (2016). Rising levels of atmospheric oxygen and evolution of Nrf2. Sci Rep..

[2] Veal E., Jackson T., Latimer H. (2018). Subcellular biochemistry. Sub Cell. Biochem..

[3] Moi P., Chan K., Asunis I., Cao A., Kan Y.W. (1994). Isolation of NF-E2-related factor 2 (Nrf2), a NF-E2-like basic leucine zipper transcriptional activator that binds to the tandem NF-E2/AP1 repeat of the beta-globin locus control region. Proc. Natl. Acad. Sci. Unit. States Am..

[4] Chan K., Lu R., Chang J.C., Kan Y.W. (1996). NRF2, a member of the NFE2 family of transcription factors, is not essential for murine erythropoiesis, growth, and development. Proc. Natl. Acad. Sci. Unit. States Am..

[5] Venugopal R., Jaiswal A.K. (1996). Nrf1 and Nrf2 positively and c-Fos and Fra 1 negatively regulate the human antioxidant response element-mediated expression of NAD(P)H:quinone oxidoreductase 1 gene. Proc. Natl. Acad. Sci. Unit. States Am..

[6] Venugopal R., Jaiswal A.K. (1998). Nrf2 and Nrf1 in association with Jun proteins regulate antioxidant response element-mediated expression and coordinated induction of genes encoding detoxifying enzymes. Oncogene.

[7] Chan K., Kan Y.W. (1999). Nrf2 is essential for protection against acute pulmonary injury in mice. Proc. Natl. Acad. Sci. Unit. States Am..

[8] Chan K., Han X.-D., Kan Y.W. (2001). An important function of Nrf2 in combating oxidative stress: detoxification of acetaminophen. Proc. Natl. Acad. Sci. Unit. States Am..

[9] Ma Q. (2013). Role of Nrf2 in oxidative stress and toxicity. Annu. Rev. Pharmacol..

[10] Dinkova-Kostova A.T., Kostov R.V., Kazantsev A.G. (2018). The role of Nrf2 signaling in counteracting neurodegenerative diseases. FEBS J..

[11] Joyce N.C. (2011). Proliferative capacity of corneal endothelial cells. Exp. Eye Res..

[12] Bourne W.M. (2003). Biology of the corneal endothelium in health and disease. Eye.

[13] Gain P., Jullienne R., He Z., Aldossary M., Acquart S., Cognasse F., Thuret G. (2016). Global survey of corneal transplantation and eye banking. Jama Ophthalmol.

[14] Kolozsvári L., Nógrádi A., Hopp B., Bor Z. (2002). UV absorbance of the human cornea in the 240- to 400-nm range. Invest. Ophthalmol. Vis. Sci..

[15] Loganathan S.K., Schneider H.-P., Morgan P.E., Deitmer J.W., Casey J.R. (2016). Functional assessment of SLC4A11, an integral membrane protein mutated in corneal dystrophies. Am. J. Physiol. Cell Physiol..

[16] Bahn C.F., Glassman R.M., MacCallum D.K., Lillie J.H., Meyer R.F., Robinson B.J., Rich N.M. (1986). Postnatal development of corneal endothelium. Invest. Ophthalmol. Vis. Sci..

[17] Tone S.O., Kocaba V., Böhm M., Wylegala A., White T.L., Jurkunas U.V. (2020). Fuchs endothelial corneal dystrophy: the vicious cycle of fuchs pathogenesis. Prog. Retin. Eye Res..

[18] Krachmer J. (1978). Corneal transplantation. Arch Ophthalmol-Chic..

[19] Soh Y.Q., Peh G.S.L., Naso S.L., Kocaba V., Mehta J.S. (2020). Automated clinical assessment of corneal guttae in fuchs endothelial corneal dystrophy. Am. J. Ophthalmol..

[20] Patel S.V., Hodge D.O., Treichel E.J., Spiegel M.R., Baratz K.H. (2020). Repeatability of scheimpflug tomography for assessing fuchs endothelial corneal dystrophy. Am. J. Ophthalmol..

[21] Rizwan M., Peh G.S., Adnan K., Naso S.L., Mendez A.R., Mehta J.S., Yim E.K.F. (2016). In vitro topographical model of fuchs dystrophy for evaluation of corneal endothelial cell monolayer formation. Adv Healthc Mater.

[22] Kocaba V., Katikireddy K.R., Gipson I., Price M.O., Price F.W., Jurkunas U.V. (2018). Association of the gutta-induced microenvironment with corneal endothelial cell behavior and demise in fuchs endothelial corneal dystrophy. Jama Ophthalmol.

[23] Liu C., Miyajima T., Melangath G., Miyai T., Vasanth S., Deshpande N., Kumar V., Tone S.O., Gupta R., Zhu S., Vojnovic D., Chen Y., Rogan E.G., Mondal B., Zahid M., Jurkunas U.V. (2019). Ultraviolet A light induces DNA damage and estrogen-DNA adducts in Fuchs endothelial corneal dystrophy causing females to be more affected. Proc. Natl. Acad. Sci. Unit. States Am..

[24] Eghrari A.O., Gottsch J.D. (2010). Fuchs' corneal dystrophy. Expet Rev. Ophthalmol..

[25] Soh Y.Q., Kocaba V., Pinto M., Mehta J.S. (2019). Fuchs endothelial corneal dystrophy and corneal endothelial diseases: east meets West. Eye Lond Engl.

[26] Mehta J.S., Vithana E.N., Tan D.T.H., Yong V.H.K., Yam G.H.F., Law R.W.K., Chong W.G.W., Pang C.P., Aung T. (2008). Analysis of the posterior polymorphous corneal dystrophy 3 gene, TCF8 , in late-onset fuchs endothelial corneal dystrophy. Investigative Opthalmology Vis Sci.

[27] Vithana E.N., Morgan P.E., Ramprasad V., Tan D.T.H., Yong V.H.K., Venkataraman D., Venkatraman A., Yam G.H.F., Nagasamy S., Law R.W.K., Rajagopal R., Pang C.P., Kumaramanickevel G., Casey J.R., Aung T. (2007). SLC4A11 mutations in Fuchs endothelial corneal dystrophy. Hum. Mol. Genet..

[28] Riazuddin S.A., Parker D.S., McGlumphy E.J., Oh E.C., Iliff B.W., Schmedt T., Jurkunas U., Schleif R., Katsanis N., Gottsch J.D. (2012). Mutations in LOXHD1, a recessive-deafness locus, cause dominant late-onset Fuchs corneal dystrophy. Am. J. Hum. Genet..

[29] Afshari N.A., Igo R.P., Morris N.J., Stambolian D., Sharma S., Pulagam V.L., Dunn S., Stamler J.F., Truitt B.J., Rimmler J., Kuot A., Croasdale C.R., Qin X., Burdon K.P., Riazuddin S.A., Mills R., Klebe S., Minear M.A., Zhao J., Balajonda E., Rosenwasser G.O., Baratz K.H., Mootha V.V., Patel S.V., Gregory S.G., Bailey-Wilson J.E., Price M.O., Price F.W., Craig J.E., Fingert J.H., Gottsch J.D., Aldave A.J., Klintworth G.K., Lass J.H., Li Y.-J., Iyengar S.K. (2017). Genome-wide association study identifies three novel loci in Fuchs endothelial corneal dystrophy. Nat. Commun..

[30] Xing C., Gong X., Hussain I., Khor C.-C., Tan D.T.H., Aung T., Mehta J.S., Vithana E.N., Mootha V.V. (2014). Transethnic replication of association of CTG18.1 repeat expansion of TCF4 gene with Fuchs' corneal dystrophy in Chinese implies common causal variant. Invest. Ophthalmol. Vis. Sci..

[31] Mootha V.V., Gong X., Ku H.-C., Xing C. (2014). Association and familial segregation of CTG18.1 trinucleotide repeat expansion of TCF4 gene in Fuchs' endothelial corneal dystrophy. Invest. Ophthalmol. Vis. Sci..

[32] Wieben E.D., Aleff R.A., Tosakulwong N., Butz M.L., Highsmith W.E., Edwards A.O., Baratz K.H. (2012). A common trinucleotide repeat expansion within the transcription factor 4 (TCF4, E2-2) gene predicts Fuchs corneal dystrophy. PLos One.

[33] Baratz K.H., Tosakulwong N., Ryu E., Brown W.L., Branham K., Chen W., Tran K.D., Schmid-Kubista K.E., Heckenlively J.R., Swaroop A., Abecasis G., Bailey K.R., Edwards A.O. (2010). E2-2 protein and Fuchs's corneal dystrophy. N. Engl. J. Med..

[34] Soh Y.Q., Peh G.S., Mehta J.S. (2018). Evolving therapies for Fuchs' endothelial dystrophy. Regen. Med..

[35] Soh Y.Q., Mehta J.S. (2018). Regenerative therapy for fuchs endothelial corneal dystrophy. Cornea.

[36] Ang M., Wilkins M.R., Mehta J.S., Tan D. (2015). Descemet membrane endothelial keratoplasty. Br. J. Ophthalmol..

[37] Azizi B., Ziaei A., Fuchsluger T., Schmedt T., Chen Y., Jurkunas U.V. (2011). p53-regulated increase in oxidative-stress--induced apoptosis in Fuchs endothelial corneal dystrophy: a native tissue model. Invest. Ophthalmol. Vis. Sci..

[38] Frausto R.F., Swamy V.S., Peh G.S.L., Boere P.M., Hanser E.M., Chung D.D., George B.L., Morselli M., Kao L., Azimov R., Wu J., Pellegrini M., Kurtz I., Mehta J.S., Aldave A.J. (2020). Phenotypic and functional characterization of corneal endothelial cells during in vitro expansion. Sci Rep-Uk.

[39] Peh G.S.L., Chng Z., Ang H.-P., Cheng T.Y.D., Adnan K., Seah X.-Y., George B.L., Toh K.-P., Tan D.T., Yam G.H.F., Colman A., Mehta J.S. (2013). Propagation of human corneal endothelial cells: a novel dual media approach. Cell Transplant..

[40] Schmedt T., Chen Y., Nguyen T.T., Li S., Bonanno J.A., Jurkunas U.V. (2012). Telomerase immortalization of human corneal endothelial cells yields functional hexagonal monolayers. PLos One.

[41] Valtink M., Gruschwitz R., Funk R.H.W., Engelmann K. (2008). Two clonal cell lines of immortalized human corneal endothelial cells show either differentiated or precursor cell characteristics. Cells Tissues Organs.

[42] Jurkunas U.V., Bitar M.S., Funaki T., Azizi B. (2010). Evidence of oxidative stress in the pathogenesis of fuchs endothelial corneal dystrophy. Am. J. Pathol..

[43] Matthaei M., Zhu A.Y., Kallay L., Eberhart C.G., Cursiefen C., Jun A.S. (2014). Transcript profile of cellular senescence-related genes in Fuchs endothelial corneal dystrophy. Exp. Eye Res..

[44] Bitar M.S., Liu C., Ziaei A., Chen Y., Schmedt T., Jurkunas U.V. (2012). Decline in DJ-1 and decreased nuclear translocation of Nrf2 in Fuchs endothelial corneal dystrophy. Invest. Ophthalmol. Vis. Sci..

[45] Suzuki T., Muramatsu A., Saito R., Iso T., Shibata T., Kuwata K., Kawaguchi S.-I., Iwawaki T., Adachi S., Suda H., Morita M., Uchida K., Baird L., Yamamoto M. (2019). Molecular mechanism of cellular oxidative stress sensing by Keap1. Cell Rep..

[46] Bryan H.K., Olayanju A., Goldring C.E., Park B.K. (2013). The Nrf2 cell defence pathway: keap1-dependent and -independent mechanisms of regulation. Biochem. Pharmacol..

[47] Liu C., Chen Y., Kochevar I.E., Jurkunas U.V. (2014). Decreased DJ-1 leads to impaired Nrf2-regulated antioxidant defense and increased UV-A-induced apoptosis in corneal endothelial cells. Invest. Ophthalmol. Vis. Sci..

[48] Kopacz A., Kloska D., Forman H.J., Jozkowicz A., Grochot-Przeczek A. (2020). Beyond repression of Nrf2: an update on Keap1. Free Radical Biol. Med..

[49] Gao Y., Yan Y., Huang T. (2014). Human age-related cataracts: epigenetic suppression of the nuclear factor erythroid 2-related factor 2-mediated antioxidant system. Mol. Med. Rep..

[50] Wakabayashi N., Itoh K., Wakabayashi J., Motohashi H., Noda S., Takahashi S., Imakado S., Kotsuji T., Otsuka F., Roop D.R., Harada T., Engel J.D., Yamamoto M. (2003). Keap1-null mutation leads to postnatal lethality due to constitutive Nrf2 activation. Nat. Genet..

[51] Malhotra D., Portales-Casamar E., Singh A., Srivastava S., Arenillas D., Happel C., Shyr C., Wakabayashi N., Kensler T.W., Wasserman W.W., Biswal S. (2010). Global mapping of binding sites for Nrf2 identifies novel targets in cell survival response through ChIP-Seq profiling and network analysis. Nucleic Acids Res..

[52] Chorley B.N., Campbell M.R., Wang X., Karaca M., Sambandan D., Bangura F., Xue P., Pi J., Kleeberger S.R., Bell D.A. (2012). Identification of novel NRF2-regulated genes by ChIP-Seq: influence on retinoid X receptor alpha. Nucleic Acids Res..

[53] Namani A., Liu K., Wang S., Zhou X., Liao Y., Wang H., Wang X.J., Tang X. (2019). Genome-wide global identification of NRF2 binding sites in A549 non-small cell lung cancer cells by ChIP-Seq reveals NRF2 regulation of genes involved in focal adhesion pathways. Aging.

[54] Katikireddy K.R., White T.L., Miyajima T., Vasanth S., Raoof D., Chen Y., Price M.O., Price F.W., Jurkunas U.V. (2017). NQO1 downregulation potentiates menadione-induced endothelial-mesenchymal transition during rosette formation in Fuchs endothelial corneal dystrophy. Free Radical Biol. Med..

[55] Miyajima T., Melangath G., Zhu S., Deshpande N., Vasanth S., Mondal B., Kumar V., Chen Y., Price M.O., Price F.W., Rogan E.G., Zahid M., Jurkunas U.V. (2020). Loss of NQO1 generates genotoxic estrogen-DNA adducts in fuchs endothelial corneal dystrophy. Free Radic. Biol. Med..

[56] Wang Z., Han N., Zhao K., Li Y., Chi Y., Wang B. (2019). Protective effects of pyrroloquinoline quinine against oxidative stress-induced cellular senescence and inflammation in human renal tubular epithelial cells via Keap1/Nrf2 signaling pathway. Int. Immunopharm..

[57] Campbell M.R., Karaca M., Adamski K.N., Chorley B.N., Wang X., Bell D.A. (2013). Novel hematopoietic target genes in the NRF2-mediated transcriptional pathway. Oxid Med Cell Longev.

[58] Itoh K., Chiba T., Takahashi S., Ishii T., Igarashi K., Katoh Y., Oyake T., Hayashi N., Satoh K., Hatayama I., Yamamoto M., Nabeshima Y. (1997). An nrf2/small maf heterodimer mediates the induction of phase II detoxifying enzyme genes through antioxidant response elements. Biochem Bioph Res Co.

[59] Jurkunas U.V., Rawe I., Bitar M.S., Zhu C., Harris D.L., Colby K., Joyce N.C. (2008). Decreased expression of peroxiredoxins in Fuchs' endothelial dystrophy. Invest. Ophthalmol. Vis. Sci..

[60] Lovatt M., Adnan K., Kocaba V., Dirisamer M., Peh G.S.L., Mehta J.S. (2019). Peroxiredoxin-1 regulates lipid peroxidation in corneal endothelial cells. Redox Biol.

[61] Dodson M., Castro-Portuguez R., Zhang D.D. (2019). NRF2 plays a critical role in mitigating lipid peroxidation and ferroptosis. Redox Biol.

[62] Alam J., Stewart D., Touchard C., Boinapally S., Choi A.M.K., Cook J.L. (1999). Nrf2, a Cap'n'Collar transcription factor, regulates induction of the heme oxygenase-1 gene. J. Biol. Chem..

[63] Hirotsu Y., Katsuoka F., Funayama R., Nagashima T., Nishida Y., Nakayama K., Engel J.D., Yamamoto M. (2012). Nrf2-MafG heterodimers contribute globally to antioxidant and metabolic networks. Nucleic Acids Res..

[64] Uchida T., Sakai O., Imai H., Ueta T. (2016). Role of glutathione peroxidase 4 in corneal endothelial cells. Curr. Eye Res..

[65] Liu C., Vojnovic D., Kochevar I.E., Jurkunas U.V. (2016). UV-A irradiation activates nrf2-regulated antioxidant defense and induces p53/caspase3-dependent apoptosis in corneal endothelial cells. Invest. Ophthalmol. Vis. Sci..

[66] Go R.-E., Hwang K.-A., Choi K.-C. (2015). Cytochrome P450 1 family and cancers. J. Steroid Biochem. Mol. Biol..

[67] Borderie V.M., Baudrimont M., Vallée A., Ereau T.L., Gray F., Laroche L. (2000). Corneal endothelial cell apoptosis in patients with Fuchs' dystrophy. Invest. Ophthalmol. Vis. Sci..

[68] Szentmry N., Szende B., Sveges I. (2005). Epithelial cell, keratocyte, and endothelial cell apoptosis in Fuchs dystrophy and in pseudophakic bullous keratopathy. Eur. J. Ophthalmol..

[69] Halilovic A., Schmedt T., Benischke A.-S., Hamill C., Chen Y., Santos J.H., Jurkunas U.V. (2016). Menadione-induced DNA damage leads to mitochondrial dysfunction and Fragmentation during rosette formation in fuchs endothelial corneal dystrophy. Antioxidants Redox Signal..

[70] Rhee S.G., Kil I.S. (2017). Multiple functions and regulation of mammalian peroxiredoxins. Annu. Rev. Biochem..

[71] Lovatt M., Adnan K., Peh G., Mehta J. (2018). Regulation of oxidative stress in corneal endothelial cells by Prdx6. Antioxidants.

[72] Jurkunas U.V. (2018). Fuchs endothelial corneal dystrophy through the prism of oxidative stress. Cornea.

[73] Benischke A.-S., Vasanth S., Miyai T., Katikireddy K.R., White T., Chen Y., Halilovic A., Price M., Price F., Liton P.B., Jurkunas U.V. (2017). Activation of mitophagy leads to decline in Mfn2 and loss of mitochondrial mass in Fuchs endothelial corneal dystrophy. Sci Rep-Uk..

[74] Miyai T., Vasanth S., Melangath G., Deshpande N., Kumar V., Benischke A.-S., Chen Y., Price M.O., Price F.W., Jurkunas U.V. (2019). Activation of PINK1-parkin–mediated mitophagy degrades mitochondrial quality control proteins in fuchs endothelial corneal dystrophy. Am. J. Pathol..

[75] Murata H., Takamatsu H., Liu S., Kataoka K., Huh N.-H., Sakaguchi M. (2015). NRF2 regulates PINK1 expression under oxidative stress conditions. PLos One.

[76] Holmström K.M., Baird L., Zhang Y., Hargreaves I., Chalasani A., Land J.M., Stanyer L., Yamamoto M., Dinkova-Kostova A.T., Abramov A.Y. (2013). Nrf2 impacts cellular bioenergetics by controlling substrate availability for mitochondrial respiration. Biol Open.

[77] Holmström K.M., Kostov R.V., Dinkova-Kostova A.T. (2016). The multifaceted role of Nrf2 in mitochondrial function. Curr Opin Toxicol.

[78] Malhotra D., Jung M., Fecher-Trost C., Lovatt M., Peh G.S.L., Noskov S., Mehta J.S., Zimmermann R., Casey J.R. (2019). Defective cell adhesion function of solute transporter, SLC4A11, in endothelial corneal dystrophies.

[79] Vilas G.L., Loganathan S.K., Quon A., Sundaresan P., Vithana E.N., Casey J. (2011). Oligomerization of SLC4A11 protein and the severity of FECD and CHED2 corneal dystrophies caused by SLC4A11 mutations. Hum. Mutat..

[80] Roy S., Praneetha D.C., Vendra V.P.R. (2015). Mutations in the corneal endothelial dystrophy–associated gene SLC4A11 render the cells more vulnerable to oxidative insults. Cornea.

[81] Guha S., Chaurasia S., Ramachandran C., Roy S. (2017). SLC4A11 depletion impairs NRF2 mediated antioxidant signaling and increases reactive oxygen species in human corneal endothelial cells during oxidative stress. Sci Rep-Uk.

[82] Korennykh A., Walter P. (2012). Structural basis of the unfolded protein response. Annu. Rev. Cell Dev. Biol..

[83] Hughes D., Mallucci G.R. (2018). The unfolded protein response in neurodegenerative disorders - therapeutic modulation of the PERK pathway. FEBS J..

[84] Engler C., Kelliher C., Spitze A.R., Speck C.L., Eberhart C.G., Jun A.S. (2010). Unfolded protein response in fuchs endothelial corneal dystrophy: a unifying pathogenic pathway?. Am. J. Ophthalmol..

[85] Okumura N., Kitahara M., Okuda H., Hashimoto K., Ueda E., Nakahara M., Kinoshita S., Young R.D., Quantock A.J., Tourtas T., Schlötzer-Schrehardt U., Kruse F., Koizumi N. (2017). Sustained activation of the unfolded protein response induces cell death in fuchs' endothelial corneal dystrophy. Investigative Opthalmology Vis Sci.

[86] Cullinan S.B., Zhang D., Hannink M., Arvisais E., Kaufman R.J., Diehl J.A. (2003). Nrf2 is a direct PERK substrate and effector of PERK-dependent cell survival. Mol. Cell Biol..

[87] Pajares M., Cuadrado A., Rojo A.I. (2017). Modulation of proteostasis by transcription factor NRF2 and impact in neurodegenerative diseases. Redox Biol.

[88] Cullinan S.B., Diehl J.A. (2004). PERK-dependent activation of Nrf2 contributes to redox homeostasis and cell survival following endoplasmic reticulum stress. J. Biol. Chem..

[89] Cullinan S.B., Diehl J.A. (2006). Coordination of ER and oxidative stress signaling: the PERK/Nrf2 signaling pathway. Int. J. Biochem. Cell Biol..

[90] Gaschler M.M., Stockwell B.R. (2017). Lipid peroxidation in cell death. Biochem Bioph Res Co.

[91] Dixon S.J., Lemberg K.M., Lamprecht M.R., Skouta R., Zaitsev E.M., Gleason C.E., Patel D.N., Bauer A.J., Cantley A.M., Yang W.S., Morrison B., Stockwell B.R. (2012). Ferroptosis: an iron-dependent form of nonapoptotic cell death. Cell.

[92] Feng H., Stockwell B.R. (2018). Unsolved mysteries: how does lipid peroxidation cause ferroptosis?. PLoS Biol..

[93] Kerins M.J., Ooi A. (2017). The roles of NRF2 in modulating cellular iron homeostasis. Antioxid. Redox Sig..

[94] Yang W.S., SriRamaratnam R., Welsch M.E., Shimada K., Skouta R., Viswanathan V.S., Cheah J.H., Clemons P.A., Shamji A.F., Clish C.B., Brown L.M., Girotti A.W., Cornish V.W., Schreiber S.L., Stockwell B.R. (2014). Regulation of ferroptotic cancer cell death by GPX4. Cell.

[95] Sun X., Ou Z., Chen R., Niu X., Chen D., Kang R., Tang D. (2015). Activation of the p62-Keap1-NRF2 pathway protects against ferroptosis in hepatocellular carcinoma cells. Hepatology Baltim Md.

[96] Roh J.-L., Kim E.H., Jang H., Shin D. (2016). Nrf2 inhibition reverses the resistance of cisplatin-resistant head and neck cancer cells to artesunate-induced ferroptosis. Redox Biol.

[97] Ishii T., Itoh K., Takahashi S., Sato H., Yanagawa T., Katoh Y., Bannai S., Yamamoto M. (2000). Transcription factor Nrf2 coordinately regulates a group of oxidative stress-inducible genes in macrophages. J. Biol. Chem..

[98] Stockwell B.R., Angeli J.P.F., Bayir H., Bush A.I., Conrad M., Dixon S.J., Fulda S., Gascón S., Hatzios S.K., Kagan V.E., Noel K., Jiang X., Linkermann A., Murphy M.E., Overholtzer M., Oyagi A., Pagnussat G.C., Park J., Ran Q., Rosenfeld C.S., Salnikow K., Tang D., Torti F.M., Torti S.V., Toyokuni S., Woerpel K.A., Zhang D.D. (2017). Ferroptosis: a regulated cell death nexus linking metabolism, redox biology, and disease. Cell.

[99] Du J., Aleff R.A., Soragni E., Kalari K., Nie J., Tang X., Davila J., Kocher J.-P., Patel S.V., Gottesfeld J.M., Baratz K.H., Wieben E.D. (2015). RNA toxicity and missplicing in the common eye disease fuchs endothelial corneal dystrophy. J. Biol. Chem..

[100] Mootha V.V., Hussain I., Cunnusamy K., Graham E., Gong X., Neelam S., Xing C., Kittler R., Petroll W.M. (2015). TCF4 triplet repeat expansion and nuclear RNA foci in fuchs' endothelial corneal dystrophy. Invest. Ophthalmol. Vis. Sci..

[101] Soragni E., Petrosyan L., Rinkoski T.A., Wieben E.D., Baratz K.H., Fautsch M.P., Gottesfeld J.M. (2018). Repeat-associated non-ATG (RAN) translation in fuchs' endothelial corneal dystrophy. Investigative Opthalmology Vis Sci.

[102] Quinti L., Naidu S.D., Träger U., Chen X., Kegel-Gleason K., Llères D., Connolly C., Chopra V., Low C., Moniot S., Sapp E., Tousley A.R., Vodicka P., Kanegan M.J.V., Kaltenbach L.S., Crawford L.A., Fuszard M., Higgins M., Miller J.R.C., Farmer R.E., Potluri V., Samajdar S., Meisel L., Zhang N., Snyder A., Stein R., Hersch S.M., Ellerby L.M., Weerapana E., Schwarzschild M.A., Steegborn C., Leavitt B.R., Degterev A., Tabrizi S.J., Lo D.C., DiFiglia M., Thompson L.M., Dinkova-Kostova A.T., Kazantsev A.G. (2017). KEAP1-modifying small molecule reveals muted NRF2 signaling responses in neural stem cells from Huntington's disease patients. Proc. Natl. Acad. Sci. Unit. States Am..

[103] Jonson I., Ougland R., Klungland A., Larsen E. (2013). Oxidative stress causes DNA triplet expansion in Huntington's disease mouse embryonic stem cells. Stem Cell Res..

[104] Kovtun I.V., Liu Y., Bjoras M., Klungland A., Wilson S.H., McMurray C.T. (2007). OGG1 initiates age-dependent CAG trinucleotide expansion in somatic cells. Nature.

[105] Paupe V., Dassa E.P., Goncalves S., Auchère F., Lönn M., Holmgren A., Rustin P. (2009). Impaired nuclear Nrf2 translocation undermines the oxidative stress response in Friedreich ataxia. PLos One.

[106] Petrillo S., D'Amico J., Rosa P.L., Bertini E.S., Piemonte F. (2019). Targeting NRF2 for the treatment of Friedreich's ataxia: a comparison among drugs. Int. J. Mol. Sci..

[107] Huang M.L.-H., Sivagurunathan S., Ting S., Jansson P.J., Austin C.J.D., Kelly M., Semsarian C., Zhang D., Richardson D.R. (2013). Molecular and functional alterations in a mouse cardiac model of Friedreich ataxia: activation of the integrated stress response, eIF2α phosphorylation, and the induction of downstream targets. Am. J. Pathol..

[108] Esteras N., Dinkova-Kostova A.T., Abramov A.Y. (2016). Nrf2 activation in the treatment of neurodegenerative diseases: a focus on its role in mitochondrial bioenergetics and function. Biol. Chem..

[109] Abeti R., Baccaro A., Esteras N., Giunti P. (2018). Novel nrf2-inducer prevents mitochondrial defects and oxidative stress in Friedreich's ataxia models. Front. Cell. Neurosci..

[110] Eghrari A.O., Vasanth S., Wang J., Vahedi F., Riazuddin S.A., Gottsch J.D. (2017). CTG18.1 expansion in TCF4 increases likelihood of transplantation in fuchs corneal dystrophy. Cornea.

[111] Soliman A.Z., Xing C., Radwan S.H., Gong X., Mootha V.V. (2015). Correlation of severity of fuchs endothelial corneal dystrophy with triplet repeat expansion in TCF4. Jama Ophthalmol.

[112] Vasanth S., Eghrari A.O., Gapsis B.C., Wang J., Haller N.F., Stark W.J., Katsanis N., Riazuddin S.A., Gottsch J.D. (2015). Expansion of CTG18.1 trinucleotide repeat in TCF4 is a potent driver of fuchs' corneal dystrophy. Invest. Ophthalmol. Vis. Sci..

[113] Eghrari A.O., Vasanth S., Gapsis B.C., Bison H., Jurkunas U., Riazuddin S.A., Gottsch J.D. (2018). Identification of a novel TCF4 isoform in the human corneal endothelium. Cornea.

[114] Kuot A., Hewitt A.W., Snibson G.R., Souzeau E., Mills R., Craig J.E., Burdon K.P., Sharma S. (2017). TGC repeat expansion in the TCF4 gene increases the risk of Fuchs' endothelial corneal dystrophy in Australian cases. PLos One.

[115] Soh Y.Q., Lim G.P.S., Htoon H.M., Gong X., Mootha V.V., Vithana E.N., Kocaba V., Mehta J.S. (2019). Trinucleotide repeat expansion length as a predictor of the clinical progression of Fuchs' Endothelial Corneal Dystrophy. PLos One.

[116] Robledinos-Antón N., Fernández-Ginés R., Manda G., Cuadrado A. (2019). Activators and inhibitors of NRF2: a review of their potential for clinical development. Oxid Med Cell Longev.

[117] Naidu S.D., Muramatsu A., Saito R., Asami S., Honda T., Hosoya T., Itoh K., Yamamoto M., Suzuki T., Dinkova-Kostova A.T. (2018). C151 in KEAP1 is the main cysteine sensor for the cyanoenone class of NRF2 activators, irrespective of molecular size or shape. Sci Rep-Uk.

[118] Ziaei A., Schmedt T., Chen Y., Jurkunas U.V. (2013). Sulforaphane decreases endothelial cell apoptosis in fuchs endothelial corneal dystrophy: a novel treatment. Invest. Ophthalmol. Vis. Sci..

